# Dance-based interventions in clinical populations: not all are the same

**DOI:** 10.3389/fpsyg.2025.1668357

**Published:** 2025-10-17

**Authors:** Michail Elpidoforou, Effie Polyzogopoulou, John T. Parissis, Dimitrios Farmakis

**Affiliations:** ^1^Interdisciplinary Laboratory for Research, Education and Disability Support - IREDS Lab, Department of Physiotherapy, University of West Attica, Athens, Greece; ^2^Department of Emergency Medicine, Athens University Hospital Attikon, National and Kapodistrian University of Athens Medical School, Athens, Greece; ^3^Department of Cardiology, Athens University Hospital Attikon, National and Kapodistrian University of Athens Medical School, Athens, Greece

**Keywords:** dance-movement psychotherapy, dance therapy, dance-movement therapy, adapted dance, inclusive dance, dance for health, therapeutic dance, dance as therapy

## 1 Introduction

Dance is described as a phenomenon in which the human body and its movement, that may have a symbolic or aesthetic value within a variable cultural and rhythmical context, are the primary tools for the human expression and communication under artistic, educational, training, recreational, therapeutic, religious, or other purposes (Basso et al., 2020; Elpidoforou, 2016; Elpidoforou et al., 2025). As a physical activity, dance motivates physical, cognitive, psychological, and social individual's integration, while it enhances neuroplasticity and functional autonomy (Dhami et al., 2014; Foster, 2013; Kattenstroth et al., 2010).

As a performing art, dance is closely related to music, since both arts have a common aspect, the dependence upon rhythm (Schrader, 2005). The therapeutic use of music concerns two main domains, Music Therapy (MT) and Music Medicine (MM), or Therapeutic Music (TM). MT refers to the psychotherapeutic use of music, while MM or TM refers to the use of music during conventional therapeutic interventions (Kang et al., 2025; Lee, 2016). Specifically, the American Music Therapy Association defines MT as “the clinical and evidence-based use of music interventions to accomplish individualized goals within a therapeutic relationship by a credentialed professional who has completed an approved music therapy program” (AMTA, 2025). Meanwhile, the British Association for Music Therapy describes it as the psychological clinical music intervention, delivered by allied healthcare professionals registered in music therapy to help people who, due to an injury, illness or disability, have a certain psychological, emotional, cognitive, physical, communicative, and social need (BAMT, 2025). MM or TM refers to the “pre-recorded music listening experiences administered by medical personnel,” who are not registered in MT (Browning et al., 2020; Lee, 2016; Lorek et al., 2023). Both approaches are performed by specially registered healthcare professionals and are indicated for several purposes in clinical practice, such as pain relief (Guetin et al., 2012; Korhan et al., 2014; Lee, 2016; Roy et al., 2012). While the use of different terms in the field of “music interventions in clinical populations” seems to be well established, this does not seem to be the case for “dance interventions in clinical populations”.

Dance can be applied in populations with various physical, cognitive, sensory, developmental or other impairments or diseases within three different contexts: (i) dance therapy (DT), dance movement psychotherapy (DMP), or dance movement therapy (DMT); (ii) adapted dance (AD), inclusive or dance for health (DfH); and (iii) therapeutic dance (TD) or dance as therapy (DaT).

As the popularity of dance-based interventions grows in healthcare, education, and community settings, it becomes essential to clarify the terminology used to describe the various approaches. This distinction is not merely semantic, but is crucial for the design of future interventional programs, the definition of their outcome measures, and the standardization in clinical, educational, community, and research settings of professional training.

## 2 Dance therapy or dance movement psychotherapy or dance movement therapy

The terms DT, DMP, or DMT (Table 1) refer to the psychotherapeutic use of movement and dance for the promotion of the emotional, cognitive, physical and social integration of an individual to enhance health and wellbeing, while its practice is facilitated by registered dance-movement therapists or psychotherapists (ADTA, 2025; Koch et al., 2019; Michels et al., 2018; Woolf and Fisher, 2015); the European Association of Dance Movement Therapy also includes the “spiritual integration” to the above list (EADMT, 2025). It is a recognized psychotherapeutic modality associated with diverse professional rights depending on national laws and regulations. There are various clinical indications, such as depression, anxiety, dementia, autism, behavior disorders, addictions, trauma, eating disorders and learning difficulties (Aithal et al., 2021; Biondo, 2023; Bucharova et al., 2020; Karkou et al., 2023, 2019; Moratelli et al., 2023; Savidaki et al., 2020).

**Table 1:** Key differences among dance-based interventions.

	**DT or DMP or DMT^a^**	**AD or ID or DfH^b^**	**TD or DaT^c^**
Primary goal	Psychotherapeutic treatment	Artistic expression, recreation, physical fitness, well-being, personal empowerment, social integration and inclusion	Improving or relieving specific disorder or disease symptoms, or medical care side effects
Facilitator	Registered in DT or DMP or DMT mental healthcare professional	Certified dance educators or instructors trained in a field of special education	Registered healthcare professionals trained in dance
Target population	Individuals with mental health needs	Individuals with disabilities or impairments	Individuals with chronic disorders or diseases
Setting	Clinical or therapeutic	Community or educational	Clinical or therapeutic
Clinical assessment and planning	Yes	No	Yes
Background	Psychodynamic, humanistic, and developmental theories	Inclusive arts education, community programs, adapted physical education, special education	Arts and physical activities as complementary non-pharmacological therapeutic tools

^a^DT, dance therapy; DMP, dance/movement psychotherapy; DMT, dance/Movement therapy.

^b^AD, adapted dance; ID, inclusive dance; DfH, dance for health.

^c^TD, therapeutic dance; DaT, dance as therapy.

The terms DT, DMP, or DMT are used interchangeably by different organizations. DMT is the term used by the American Dance Therapy Association (ADTA, 2025) and the Dance Movement Therapy Association of Australasia (DTAA, 2025), while DMP is the term used by the Association for Dance Movement Psychotherapy of United Kingdom (ADMPUK, 2025). DT is more commonly used in research (Barcelos de Souza et al., 2025; Tomaszewski et al., 2023) and, along with DMT and DMP, refers to the same intervention, although there is not a unique accepted universally name for it (Dunphy et al., 2021).

National dance therapy associations have outlined several pathways for becoming a dance-movement therapist or psychotherapist, ranging from private institutions to university PhD programs (Dunphy et al., 2021). A minimum of 2 years of full-time training is required, including personal psychotherapeutic treatment, clinical training, fieldwork, internships, and supervision, to become a registered dance-movement therapist or psychotherapist (Dunphy et al., 2021).

## 3 Adapted or inclusive dance or dance for health

The terms AD, ID or DfH (Table 1) refer to a dance program appropriately modified for individuals with movement, sensory, cognitive, developmental or other impairments for their recreation, inclusion, social integration, as well as the enhancement of their health, well-being and physical fitness; the program is delivered by certified dance educators or instructors experienced or trained in a field of special education (Côté-Séguin, 2020; Joung et al., 2024; Lentzari et al., 2023; McGuire et al., 2019; Mendoza-Sanchez et al., 2022). The term Psychomotor dance-based exercise (Guzman-Garcia et al., 2013), has also been used in the literature interchangeably with the above terms. AD, ID or DfH programs are designed to be inclusive, allowing individuals of varying abilities or disabilities to participate in dance practices and enhance their physical activity levels (Atkins et al., 2018; Frable et al., 2025; Schroeder et al., 2017). These programs often focus on the joy of movement, fostering creativity and expression, and building community and self-efficacy among participants (Bungay et al., 2022; Bungay and Jacobs, 2020; McRae et al., 2018). Although they may have therapeutic effects, they are not structured as formal therapy sessions nor include adaptations to meet specific clinical needs, and do not require conduction by licensed therapists (Waugh et al., 2024).

These interventions focus on facilitating an inclusive and creative learning process for individuals of different abilities or disabilities in educational or community settings (Dinold and Zitomer, 2015). Suggested practices include adapting and modifying dance techniques and teaching methods, using inclusive language in instruction, encouraging participation, and fostering social interaction in an individualized, team-oriented approach (Dinold and Zitomer, 2015). Numerous AD, ID or DfH programs (Massó-Guijarro et al., 2025; McGuire et al., 2019), suggested guides for developing an inclusive dance intervention (Duarte Machado et al., 2025), and teacher training programs (ALLPlayDance, 2025; Candoco, 2025; DanceAbility, 2025; ParableDance, 2025; Seedbed, 2025; SENDance, 2025; SENDTraining, 2025) exist. However, the lack of clear professional standards and of a representative international organization highlight the need for the development of a unified framework for these interventions.

## 4 Therapeutic dance or dance as therapy

The term TD or DaT (Table 1) is often used in the scientific literature in different contexts without having yet been clearly defined. As the most loosely defined category, it encompasses structured dance interventions designed to promote health and wellbeing aspects of clinical populations without necessarily meeting the criteria of DT, DMP, or DMT. DaT, as a term, is used to define dance interventions in clinical populations conducted by healthcare practitioners who are not dance-movement therapists (Tomaszewski et al., 2023). Examples include studies in patients with hypertension (Peng et al., 2024, 2021; Wang et al., 2024), heart failure (Gomes Neto et al., 2014), breast cancer (Karkou et al., 2021), Parkinson's disease (Elpidoforou et al., 2022), multiple sclerosis (Davis et al., 2023), chronic pain (Hickman et al., 2022), Alzheimer's disease (Ruiz-Muelle and Lopez-Rodriguez, 2019), and human immunodeficiency virus (Morgan, 2014).

There are many studies in which the terms DT or DMP or DMT have been used—instead of TD or DaT—to describe a dance program applied to clinical populations with specific therapeutic endpoints, but not using specific DT/DMP/DMT assessment or therapeutic tools, or not being facilitated by appropriate therapists. This leads to spurious conclusions concerning the possible effects of each dance intervention, and it does not allow proper use in clinical and research settings.

Thus, TD or DaT could be defined as the complementary non-pharmacological therapeutic use of dance for the improvement or relief of symptoms related to a disorder or disease–not necessarily mental – or symptoms that appear as side effects of medical care, and is facilitated by registered healthcare practitioners trained in dance (Bognar et al., 2017; Brito et al., 2021; Bruyneel, 2019; Elpidoforou et al., 2022; Karkou et al., 2021). The above definition is closely related to the existing definition of TM or MM, that differentiates it from MT (Browning et al., 2020; Kang et al., 2025; Lee, 2016; Lorek et al., 2023), as previously stated. According to that, both TM or MM and TD or DaT are complementary, non-pharmacological, arts-based interventions delivered by registered healthcare practitioners in clinical contexts for non-psychotherapeutic purposes.

These interventions aim to use dance as a complementary non-pharmacological therapeutic tool in clinical settings, facilitated by healthcare practitioners with non-psychotherapeutic training backgrounds (Tomaszewski et al., 2023). According to the above, the context determines if the purpose of a dance intervention is basically psychotherapeutic, therapeutic for specific disorder or disease symptoms, or non-therapeutic. Thus, several programs and teacher training programs could be delivered as TD or DaT interventions under the appropriate context (DfPD, 2025; McRae et al., 2018; WSU, 2025). However, no professional standards have been clearly established yet. The development of an appropriate consensus on these standards could clarify the role of TD or DaT professionals.

## 5 Discussion and future directions

Each of the three different dance-based intervention categories contributes uniquely to human health and experience and may offer profound benefits across diverse populations. However, clarity in terminology is crucial for refining practice, advancing research, and ensuring ethical service delivery. Recognizing the differences among these approaches allows us to better harness their potential.

The question that arises is what practical recommendations could be derived from the above clarified definitions. As noted above, while content matters, context plays the principal role in the case of dance interventions for clinical populations. Each dance intervention may have a psychotherapeutic purpose, a complementary therapeutic purpose targeting specific disease or disorder symptoms, or a non-therapeutic purpose. Accordingly, the primary aim and the facilitator's professional background determine the category to which a given dance intervention belongs. Thus, content seems to serve context and not vice versa. We recommend the use of the terms DT or DMP or DMT in the case of a mental health care professional registered in DT or DMP or DMT and in the context of a primary psychotherapeutic goal. Instead, we recommend the use of the terms AD or ID or DfH in the case of a certified dance educator or instructor with a further training in a field of special education and in the context of a primary goal of artistic expression, recreation, physical fitness, well-being, personal empowerment, or social integration and inclusion. Finally, we recommend the use of the terms TD or DaT in the case of a registered healthcare practitioner with additional training in dance in the context of facilitating a dance intervention with a primary goal of improving or relieving specific disorder or disease symptoms, or medical care side effects.
